# A Comparative Study of the Microleakage of Resilon/Epiphany and Gutta-Percha/AH-Plus Obturating Systems

**Published:** 2012-08-01

**Authors:** Elmakki Fathia, Neamat Hassan Abu-bakr, Ibrahim Yahia

**Affiliations:** 1. Conservative Dentistry Division, Department of Oral Rehabilitation, Dental School, University of Khartoum, Sudan

**Keywords:** Adhesive Dental Materials, Ah Plus, Endodontic Materials, Leakage, Gutta-Percha, Resilon, Epiphany, Root Canal Obturation, Root Canal Therapy

## Abstract

**Introduction:**

The aim of the present study was to investigate and compare the apical sealing ability of Resilon/Epiphany-filled root canals with those that were obturated with gutta-percha/AH-Plus endodontic sealer.

**Materials and Methods:**

A total of 60 extracted human single-rooted teeth were selected; 25 teeth for each test group and five for each control group. After conducting conventional endodontic treatment, the teeth were immersed in physiologic saline solution for thirty days, and subsequently sealed and stored in methylene blue dye solution for seven days. The teeth were sectioned to evaluate the linear apical leakage using a stereoscopic microscope. The data were statistically analyzed by non-parametric Kruskal-Wallis and Mann-Whitney U tests.

**Results:**

The results showed significant differences between the two groups of endodontic sealers (P<0.001).

**Conclusion:**

Within the limitation of the present in vitro study, Resilon/Epiphany sealer had better apical sealing ability than gutta-percha/AH-Plus sealer.

## Introduction

The main objectives of root canal treatment are to clean and shape the root canal, removing all organic material, and to seal the pulp chamber and canal. Success in endodontic treatment is predominantly determined by complete obturation of the canal system. Gutta-percha, which has been commonly used for this purpose, has universally been accepted as the gold standard for root canal filling materials. However, it fails to provide an adequate apical seal, as it lacks the ability to bond or adhere to both the root canal dentine and sealer [1][2][3][4][5]. Thus, finding a substitute that offers a superior seal of the root canal system has become a challenge in modern Endodontics. Many different materials have been proposed as root canal fillings. The adhesion of endodontic sealers to both the obturation material and to dentin may improve their sealing properties [6]. Resin-bonded root canal filling materials have been suggested as an alternative to the traditional gutta-percha based system to obtain a better seal. Resilon has been developed to replace gutta-percha and traditional sealers for root canal obturation. Preliminary studies of the Resilon/Epiphany adhesive root-filling materials have shown remarkable promise, i.e. a decrease in the amount of leakage when compared with conventional gutta-percha fillings [7][8][9], and an improvement in the root fracture resistance was shown [10]. The aim of the present study was to investigate and compare the apical sealing ability of Resilon/Epiphany-filled root canals with those that were obturated with gutta-percha/AH-Plus endodontic sealer by conducting apical leakage using methylene blue dye penetration test.

## Materials and methods

A total of sixty newly extracted single-rooted human teeth were used in this study. All selected teeth were free from any open apices, cracks, and resorptive defects. Teeth were carefully cleaned with curettes to remove the soft-tissue remnants and stored in saline solution prior to instrumentation. The crowns of the teeth were sectioned at the cemento-enamel junction by using water-cooled diamond discs.

The canal length was visually established by placing a size 15 K-type file (Kerr, Romulus, MI, USA) into each root canal until the tip of the file was visible at the tip of the apical foramen. The working length was established 1mm short of the apex. Cleaning and shaping were performed. The root canals were prepared with K-flexofile (Dentsply, Maillefer, Ballaigues, Switzerland), with the crown-down technique until the working length was reached. Instruments were adjusted to the walls of the canals, followed by instruments in ascending order of diameter, to achieve an apical flare equal to that provided by the size 45 file. During the biomechanical preparation, the canal patency was maintained with a size 10 file and irrigation with 3% sodium hypochlorite (NaOCl) solution (Nice chemicals Pvt. Ltd., India) was carried out. After preparation the canals were instrumented with size-10 and -15 files 2 mm beyond the foramen to standardize the foramina diameter. After using the last file, the roots were irrigated with physiologic saline solution and dried with absorbent paper points (Dentsply, Konstanz, Germany). The canals were irrigated between instrumentation with 17% ethylene diamine tetra-acetic acid (EDTA) (Prev, LTD, India) and 3% NaOCl. EDTA was used as the final rinse before root canal obturation, as required by the manufacturer of Epiphany to remove the smear layer. To remove the remaining NaOCl and EDTA solutions final irrigation was performed with 5 mL normal saline and dried with a paper point. This protocol was employed for all teeth to eliminate the introduction of an additional experimental variable. The debrided root canals were dried with multiple paper points and randomly divided into two experimental groups (25 for each group) and two control groups (five for each group).

The teeth of group I were obturated using the lateral condensation technique using gutta-percha master point (Hygenic, Coltene, Germany) dipped in AH sealer (Dentsply, De Trey, Konstanz, Germany). Excess gutta-percha was removed and condensed with plugger 1 mm below the canal opening; the root canal opening was sealed with a ready-made temporary filling material Cavit (ESPE, Seefeld, Germany). In group II before inserting the sealer, the self-etching primer (Epiphany primer) was introduced into the root canal with microbrush and after 30 seconds, the excess material was removed with absorbent paper points. Teeth were obturated using the lateral condensation technique, a pre-measured master point to fit the diameter and length of the root canal, coated with the sealer (Epiphany Root Canal Sealant) was placed in the root canal and condensed with a finger spreader. The rest of the canal was filled with accessory points dipped in a small amount of sealer, which were polymerized for 40 seconds in the coronal portion. The excess points were removed with a hot instrument; the root canal opening was sealed with a ready-made temporary filling material (Cavit).

The tooth roots in group III (positive control) were not obturated and were used to demonstrate dye leakage throughout the entire length of the canal. The coronal third was filled with Cavit. The root canals in group IV (control) were obturated with gutta-percha/AH-Plus. Finally, all the teeth were immersed in physiologic saline solution for 14 days. The solution was renewed each week.

### Evaluation of Apical Leakage

Apical leakage was estimated using a dye penetration test. The root surfaces were covered with two layers of nail varnish and sticky wax with the exception of the apical 1 mm, but the root surfaces of the group IV teeth were entirely coated with two layers of nail varnish and sticky wax to prevent possible leakage. The teeth were then placed into 2% methylene blue dye solution for seven days at 37ºC. After being removed from the dye solution, the teeth were washed with water and dried, and then the nail varnish and sticky wax were removed with a scalpel. The teeth were sectioned longitudinally in a bucco-lingual direction through the center of the root. Linear apical leakage was measured from the apex to the coronal extent of the methylene blue dye penetration. Maximum dye penetration was measured in millimeters in each half of each tooth from the apex to the most coronal part of the root canal to which the dye had penetrated usinga stereomicroscope at 25× (Olympus BX50, Japan). The results were collected, tabulated and statistically analyzed using non-parametric Kruskal-Wallis and Mann-Whitney U tests. The level of significance was fixed at P<0.05.

## Results

**Figure 1:**
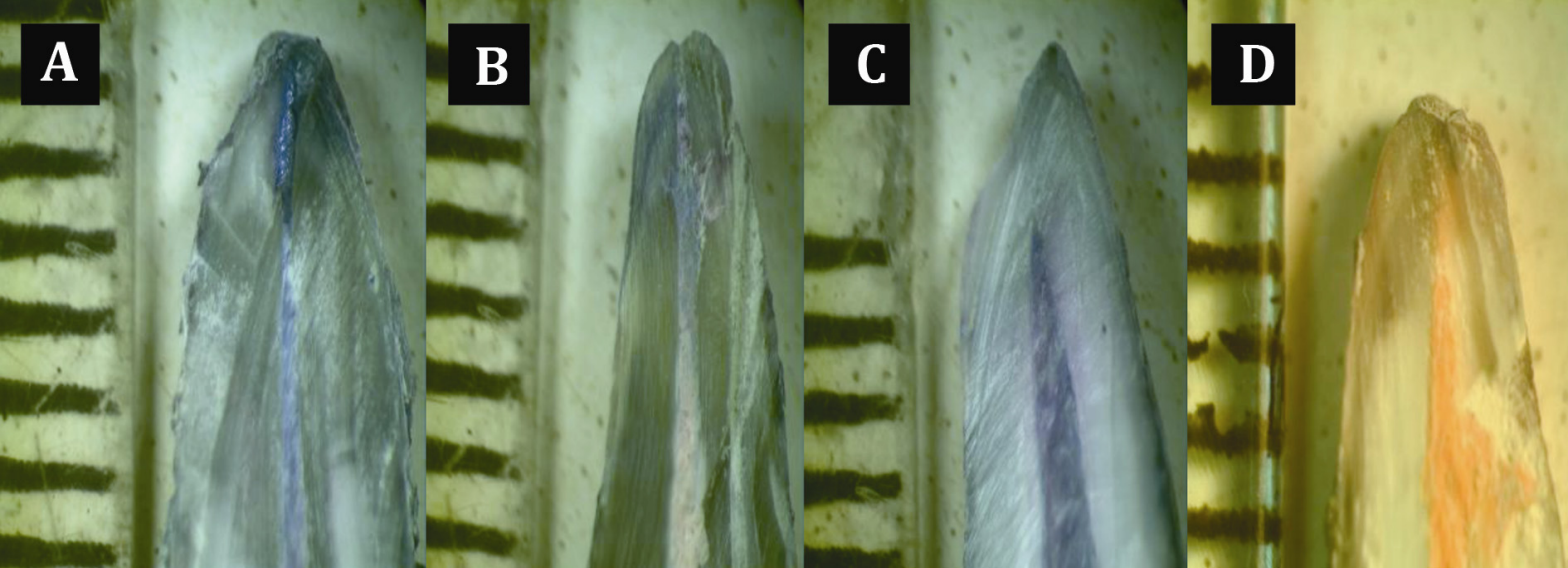
A) Dye Penetration showing extent of apical leakage in group I (gutta-percha/AH plus); B) Dye Penetration showing extent of apical leakage in group II (Resilon/Epiphany); C) Large apical leakage in group III; D) No dye penetration in group IV

Means and standard deviations for the degree of leakage of the experimental groups were measured (Table 1). Dye penetration in the experimental groups occurred mainly at the interface of the sealer and the root canal wall in the majority of the teeth. Figure 1 shows the degree of microleakage among all tested materials. The data obtained evaluating the apical seal was subjected to statistical analysis using Non-parametric Mann-Whitney U test revealed that group II (Resilon and Epiphany) had the least amount of microleakage compared to group I (gutta-percha and AH-Plus) which proved to have a significant amount of microleakage (P<0.001). Group III teeth showed complete leakage throughout the length of the root canal, whereas group IV teeth revealed no dye penetration. The computed value of P was less than 0.05, which indicates a statistically significant difference between the groups of our study.

**Table 1:** Mean (SD) microleakage of the examined groups

**Groups**	**Mean (SD)**
**Group I (Gutta-percha/AH plus)**	2.53 (1.19)
**Group II (Resilon/Epiphany)**	1.06 (0.78)
**Group III (Positive Control)**	12.5 (5.25)

## Discussion

Complete obturation of the root canal system with impervious, biocompatible, and dimensionally stable filling-material is essential for successful root canal treatment. However, it has been reported that a complete seal of the root canal system is almost impossible with currently accepted materials and techniques using a combination of gutta-percha and root canal sealer [5]. Ideally, the root canal sealer should be capable of producing a bond between the core material and the root dentine, effectively preventing leakage [11]. Three-dimensional sealing of the root canal is one of the main goals of endodontic treatment and is essential for preventing re-infection of the canal and for preserving the health of the periapical tissues, thereby ensuring the success of root canal treatment. Thus, several types of endodontic sealers have been recommended to achieve this goal and, consequently, the evaluation of the apical sealing ability of the sealers is important. It should be noted that the coronal seal is of equal importance to the apical seal of the root canal for the success of endodontic treatment [12].

Leakage studies on the sealing properties of endodontic materials are still important and relevant [13]. Different methods have been used to evaluate the sealing of endodontic materials. Assessment of linear dye penetration is a common method used to explore apical leakage of root fillings after splitting the roots. The leakage marker used in this study was methylene blue because it has a low molecular weight and penetrates more deeply along the root canal filling [14]. It is well known that root-filling materials penetrate better into dentinal tubules in the absence of the smear layer [15]. In the present investigation, 17% EDTA and 3% NaOCl were used as irrigation solution to remove the smear layer [1]. In the present study, none of the root canal filling materials and sealers exhibited complete apical sealing. Resilon/Epiphany resulted in significantly less apical leakage measured by the dye extraction method than the combination of gutta-percha/AH-Plus sealer. Present results agree with the results obtained by Shipper et al., who concluded that the Resilon/Epiphany root canal filling system exhibited the least leakage [7]. In a comparative study of sealing ability of MTA, Resilon and gutta-percha, Froughreyhani et al. showed that MTA was superior to Resilon, and Resilon had better sealing ability than gutta-percha [16]. Bodrumlu and Tunga [17] and Kqiku et al. [18]) drew similar conclusions using leakage tests and found that Epiphany and Resilon were superior to gutta-percha.

On the other hand, other studies using bacterial leakage tests as well as a dye penetration method, did not observe a difference between Resilon and gutta-percha [19][20]. Kangarlou et al. recently conducted a study on the bacterial leakage of root canals sealed with GuttaFlow, Resilon/Epiphany, or gutta-percha/AH26. They showed no significant difference between the tested materials [21]. Moreover, a recent study concluded that Resilon/Epiphany is not superior to gutta-percha and conventional epoxy resin sealer [22]. Discrepancies between the results of these and other [23][24][25][26][27] leakage studies could be because of differences in the methodology used for microleakage evaluation. Clinical data is required to provide more sound evidence to support either argument.

## Conclusion

Within the limitation of the present study, Resilon/Epiphany sealer had better apical sealing ability than gutta-percha/AH-Plus sealer.
